# Sleep Duration and Quality in Adolescents: Associations With Suicidal Ideation

**DOI:** 10.1002/jad.12473

**Published:** 2025-01-25

**Authors:** Theresa Lemke, Sebastian Hökby, Vladimir Carli, Gergö Hadlaczky

**Affiliations:** ^1^ National Centre for Suicide Research and Prevention (NASP), Department of Learning, Informatics, Management and Ethics (LIME) Karolinska Institutet Stockholm Sweden; ^2^ National Centre for Suicide Research and Prevention (NASP), Centre for Health Economics, Informatics and Health Services Research (CHIS) Stockholm Health Care Services Stockholm Sweden

**Keywords:** adolescents, depression, mental health, sleep, suicidal ideation

## Abstract

**Introduction:**

Inadequate sleep duration and sleep‐related problems are highly prevalent among adolescents and pose a significant health risk during a critical development stage. This study seeks to explore associations between sleep and suicidal ideation among adolescents.

**Methods:**

Cross‐sectional questionnaire data from the baseline wave (2016‐2018) of a cohort of 12‐ to 16‐year‐old Swedish adolescents (*n* = 4433, 50.39% girls) were analyzed. A split‐sample approach was used for exploratory analyses and model selection. Logistic regression was used to estimate the associations between suicidal ideation and self‐reported sleep parameters (weekday sleep duration, sleep quality), both adjusted and unadjusted for depression.

**Results:**

Adolescents with suicidal ideation slept on average 60 min less on weekdays and reported worse sleep quality compared to those without suicidal ideation. Suicidal ideation was significantly associated with weekday sleep duration (*p* = 0.0267) and self‐perceived sleep quality (*p* = 0.0003). Associations remained after controlling for depression.

**Conclusions:**

Sleep problems in adolescents are associated with suicidal ideation, beyond the effect of depression. Findings may have implications for screening and suicide prevention among clinical populations of adolescents, as well as for public health interventions aimed at promoting sleep and mental health in adolescents.

## 1 Introduction

Inadequate sleep and sleep‐related problems are prevalent health concerns among adolescents. Many adolescents do not receive the recommended number of hours of sleep on weekdays (Chaput and Janssen 2016; Galan‐Lopez et al. 2021; Lemke et al. 2023; Wheaton et al. 2018) and frequently experience poor sleep quality, such as having problems falling asleep, waking up during the night, and not feeling rested in the morning (Galan‐Lopez et al. 2021). Adolescence stands out as a critical period for the development of sleep‐related problems. Physiological changes result in a delay of adolescents' sleep‐wake cycle and their natural “clock” regulating sleep and wakefulness, also known as the chronotype (Hagenauer et al. 2009). As a result, adolescents tend to stay awake longer in the evening and fall asleep at later times (Colrain and Baker 2011). These disruptions in sleep habits are often further exacerbated by increasing use of electronic media and exposure to social and emotional stressors commonly experienced during adolescence (Carskadon 2011).

Sleep‐related problems during adolescence have severe implications for adolescents' physical health, cognitive development, academic performance, safety, and mental health (Owens et al. (2014); Tarokh, Saletin, and Carskadon 2016). With regards to the impact on mental health, studies have shown that sleep problems are associated with higher risks for mental health problems such as depression and anxiety (Lovato and Gradisar 2014; Orchard et al. 2020; Short et al. 2020). Insufficient sleep is also recognized to be associated with impaired cognitive and psychological functioning, for example, executive functioning, emotion regulation, and affective reactivity (Baum et al. 2014; Gruber et al. 2023; McMakin et al. 2016). Deficits in these functions appear to carry an increased risk for adolescents to engage in suicidal thoughts and behaviors (Chiang, Chen, and Chou 2023; Miranda et al. 2012). This raises the question whether impaired sleep constitutes a possible pathway for the occurrence of suicidal ideation and whether this pathway exists independent of the presence of symptoms of psychopathologies such as depression.

Previous studies suggest that sleep‐related problems, including insufficient sleep duration and poor sleep quality are associated with an increased risk of suicidality in adolescents (Goldstein and Franzen 2022; Kearns et al. 2020; J.‐W. Liu et al. 2019). However, the existing studies have some limitations that the present study aims to address. Firstly, although sleep ought to be considered a multidimensional phenomenon (Buysse 2014), most studies have only investigated the effect of single sleep variables (e.g., sleep duration) on suicide risk. This study aims to investigate different sleep parameters and their incremental contributions to the effect of suicidal ideation. Secondly, existing studies often conflate different suicidality outcomes (i.e., suicidal ideation, suicide attempts, plans, death by suicide), despite research showing that these are distinct concepts that may be influenced differently by certain risk factors. Finally, few of the studies investigating the sleep‐suicidal ideation relationship adjust for depression as a covariate. Given the available evidence on depression being an established risk factor of suicidality (Carballo et al. 2020), not accounting for the effect of depression limits the interpretability of the results in terms of the direct effect of sleep on suicidal ideation. This study aims to address these limitations by investigating the relationship between different sleep parameters and suicidal ideation in adolescents, using validated measurement tools for suicidal ideation and sleep and adjusting for the effect of depression.

Suicidality is a phenomenon involving a large number of different and interrelated risk factors (Franklin et al. 2017). The present study investigates various sleep outcomes using Goldstein and Franzen (2022) framework. According to this model, the relationship between sleep and suicidality can occur through both proximal and distal processes. Proximal processes include bi‐directional associations between sleep and emotional‐ and behavioral dysregulation. At the same time, poor sleep health is associated with distal psycho‐social risk factors for suicidality, for example, psychopathologies such as depression. This study utilizes the framework by Goldstein and Franzen (2022) for studying the relationships between different indicators of sleep health and suicidal ideation, while adjusting for the relationship between depression and sleep, and suicidality respectively. As highlighted by the framework as well as the available literature described above, depression as a distal factor may influence and interact with sleep problems and suicidal thoughts. In this study we try to understand the unique contribution of sleep, if any, by adjusting for the potential confounding effect of depression. We also examine different constructs related to sleep such as *duration*, *quality* and *timing* considering that previous research suggests that these may have differential effects on health (Buysse 2014).

This study builds on a previously published study about associations between different sleep variables and depression using a large community‐based representative sample of Swedish adolescents (Lemke et al. 2023) and aims to contribute to the understanding of sleep as a modifiable risk factor and potential entry point for early identification of at‐risk individuals and suicide‐preventive interventions in adolescents.

## 2 Materials and Methods

This study analyzed cross‐sectional baseline data from a cohort of 12‐16‐year‐old adolescents in Stockholm County, Sweden. A detailed account of the study design, data collection process, and measures can be retrieved from a previous publication presenting findings about the relationship between sleep and depression (Lemke et al. 2023). This study further employed a “split‐sample” approach, randomly dividing the total sample (*n* = 10,288) into an exploratory sample (*n* = 5145) used for model selection and a validation sample (*n* = 5143) used for conducting the analyses that are presented in this report (the difference in sample size is due to the sample‐splitting being stratified by suicidal ideation). This approach aims to reduce the risk of false‐positive findings and overfitting (Lever, Krzywinski and Altman (2016); Lubke and Campbell 2016) and was chosen to support a more rigorous analysis process given that the present study constitutes an extension of previously published analyses using the same dataset (Lemke et al. 2023).

### 2.1 Study Population and Recruitment

Baseline data was collected between August 2016 and November 2018 at 116 elementary and junior high schools, with students attending grades 7 or 8 during baseline data collection. The total sample was *n* = 10288, after the exclusion of participants that were either younger or older than the age range of 12–16 years. In the present study, findings are presented for the validation sample, including participants with complete cases from the regression analysis (*n* = 4433). Sample characteristics for both the exploratory sample and the validation sample are displayed in Table 1.

**Table 1:** Sample characteristics.

Variable	Total sample (n = 10,288)		Exploratory sample (n = 4460)	Validation sample (n = 4433)
	**M (SD)**	**Missings, n (%)**	**N (%)**	**N (%)**
**Gender**				
Boys (coded as =0)	5179 (50.34)	89 (0.87)	2218 (49.73)	2199 (49.61)
Girls (coded as =1)	5020 (48.79)		2242 (50.27)	2234 (50.39)
			**M (SD)**	**M (SD)**
**Age** (12–16 years; discrete variable)	13.98 (0.72)	31 (0.30)	13.99 (0.727)	13.97 (0.715)
**Socio‐economic status** (range: 1‐5; higher scores indicate self‐perception as “wealthier”)	4.47 (0.86)	57 (0.55)	4.51 (0.816)	4.50 (0.839)

*Notes:* M = Mean, SD = Standard deviation. Exploratory sample: Sub‐sample of the total sample used for model selection, complete cases (variables in the selected regression model) (*n* = 4460). Validation sample: Sub‐sample of the total sample for which the final regression model was fitted, and results are presented in this article, complete cases (variables in final regression model) (*n* = 4433).

Written consent was obtained from adolescents or from a legal guardian if the participant was under the age of 15. The study received ethical approval by the regional ethics committee in Stockholm (2016/2175‐31/5).

### 2.2 Measures

Sleep habits were measured using the Karolinska Sleep Questionnaire (KSQ) (Nordin, Åkerstedt, and Nordin 2013). Sleep duration on weekdays and weekends was calculated from items asking participants to report their bedtime, sleep onset latency and wake time separately for weekdays and weekends (Lemke et al. 2023). Sleep quality was measured as the average score across six items from the KSQ measuring different sleep‐related problems, such as difficulties falling asleep, repeated awakenings, and not feeling well‐rested after waking up (Nordin, Åkerstedt, and Nordin 2013). The mean sleep quality score indicates the average frequency of experiencing these difficulties during the past 3 months, with higher scores indicating better sleep quality (lower frequency of reported problems). Chronotype was operationalized as the sleep debt‐corrected midpoint of sleep on weekends (Roenneberg et al. 2004, 2019), calculated based on the sleep habits reported in the KSQ (see Lemke et al. 2023 for a detailed description of this approach).

Suicidal ideation was measured using an item from Paykel's suicide scale (Paykel et al. 1974): *“Have you, in the past 2 weeks, reached a point where you seriously considered taking your own life or even planned how you would go about doing it?”*. Participants who reported suicidal thoughts “sometimes,” “often,” “very often” and “always” were categorized as having suicidal ideation, whereas participants answering “never” or “seldomly” were categorised as not having suicidal ideation (Wasserman et al. 2015).

Covariates were selected for the exploratory analysis based on theoretical and empirical considerations.

Depression was included as a covariate due to its potential confounding effect on the relationship between sleep problems and suicidal ideation (Alvaro, Roberts, and Harris 2013; Carballo et al. 2020; also described in the framework by Goldstein and Franzen 2022), enabling to assess the effect of sleep on suicidal ideation beyond associations with depression. Depression was measured using the Beck Depression Inventory‐II (BDI‐II) (Beck, Steer, and Brown 2005; Wang and Gorenstein 2013). As described by Lemke et al. (2023), items relating to sleep and sexual interest were excluded before calculating participants' total BDI‐II scores. The sleep item was excluded because it is dependent on the main predictor variables; sexual interest due to poor item loading; and additionally, for the present study, the item on suicidal ideation was excluded because it is dependent on the outcome variable. A dichotomous depression variable was calculated, with BDI scores > 13 indicating the presence of clinical symptoms of depression (Beck, Steer, and Brown 2005).

The following socio‐demographic variables were included in the analyses as covariates to account for socio‐demographic differences in both prevalence of suicidal ideation (Biswas et al. 2020) and sleep habits and sleep‐related problems (Chaput and Janssen 2016; Galan‐Lopez et al. 2021; Lemke et al. 2023): gender (male, female), age, and self‐reported socio‐economic status (score from 1 to 5, higher scores indicating socio‐economic status perceived as “wealthier”).

### 2.3 Analyses

The exploratory sample was used for developing and selecting the final regression model. For this purpose, variables were included in the exploratory analyses using a hierarchical logistic regression model with the following levels: (1) socio‐demographic variables (gender, age, self‐perceived socio‐economic status), (2) sleep duration (weekdays, weekends), (3) sleep quality, and (4) chronotype. Model fit of the increasingly more complex models was assessed through Likelihood‐ratio tests. The final selected regression model included socio‐demographic variables as well as statistically adjusted yet still significant sleep variables (weekday sleep duration and sleep quality). In the exploratory sample, weekend sleep duration and chronotype were not significantly associated with suicidal ideation and did not improve the model fit. To maintain a parsimonious model, these variables were not included in the final regression model.

The final regression model was then fitted to the validation sample (*n* = 4433, complete cases for regression variables) and generated the main findings reported in this paper. Furthermore, a regression model is presented after adjusting for the effect of depression, to show the direct effect of sleep on suicidal ideation, that is not explained by depression. To account for the nested data structure (school clusters, *n* = 116) and ensure precise confidence intervals, cluster‐robust standard errors were used (Zeileis 2006). A regression model investigating a potential moderation effect of depression (including interaction terms between sleep variables and depression) was fitted but showed non‐significant moderation effects (see Appendix 1). To account for potential between‐school differences in suicidal ideation, a mixed‐effects model (random intercept) was fitted, which showed similar relationships between sleep and suicidal ideation as the main analysis described above (see Appendix 2).

## 3 Results

**Figure 1:**
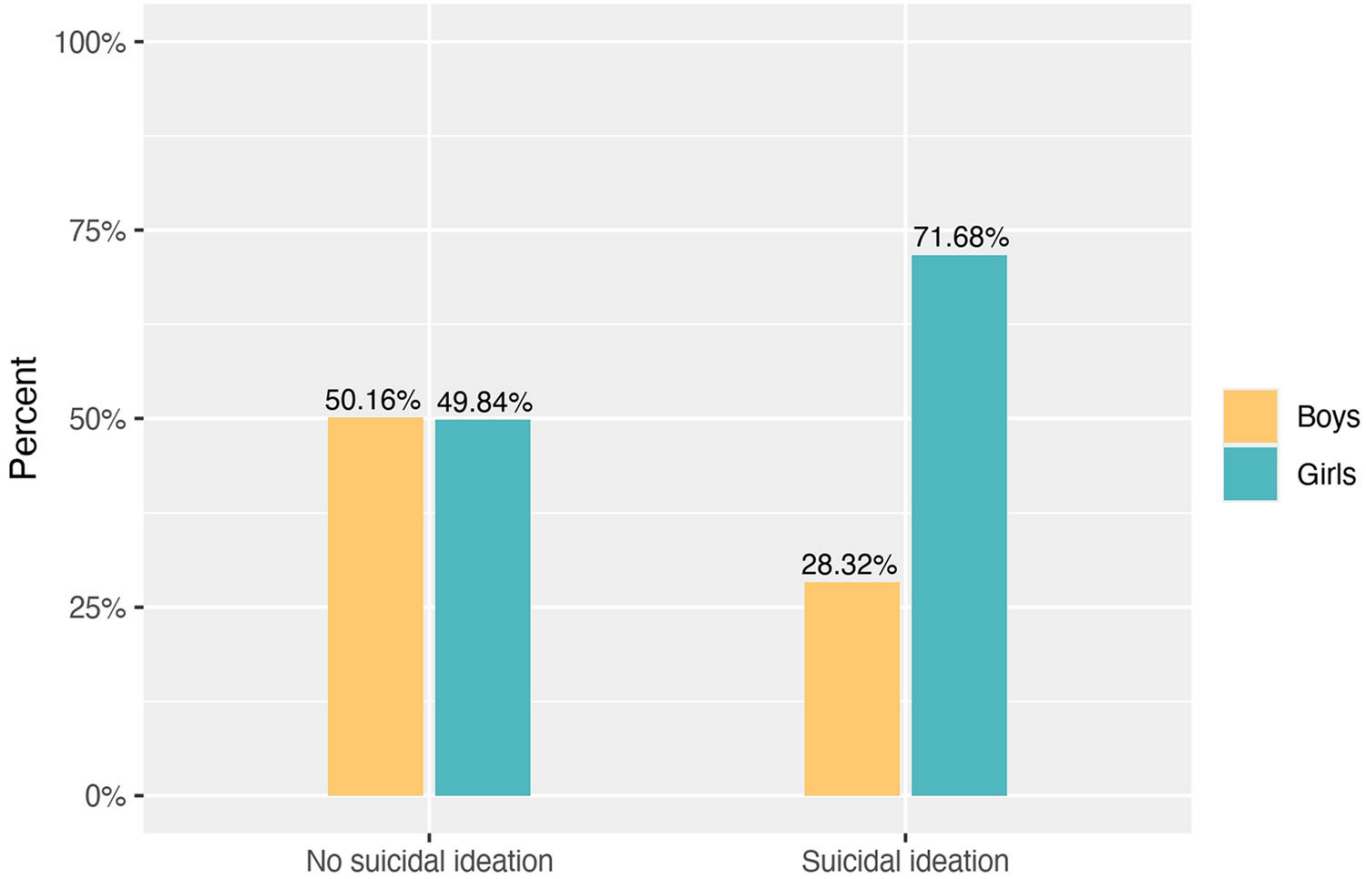
Adolescents with and without suicidal ideation, gender distribution (%).

Suicidal ideation was prevalent in 2.55% (*n* = 113) of adolescents in this sample (validation sample, *n* = 4433), with 71.68% of those being girls (Figure 1).

Adolescents with suicidal ideation slept on average 6:54 h (SD = 1:31 h) on weekdays, compared to 7:54 h (SD = 1:11 h) among those without suicidal ideation. Among adolescents with suicidal ideation, 75.22% slept less than the recommended amount of 8 h on weekdays, while this proportion was 45.40% among those without suicidal ideation. Among adolescents with depression, those who also had suicidal ideation slept on average 6:44 h (SD = 1:31) compared to 7:17 h (SD = 1:22) among those with depression but no suicidal ideation.

Difficulties relating to sleep quality (“often” or more frequently during the previous 3 months) were more prevalent among adolescents with suicidal ideation (53.10%), compared to those without suicidal ideation (12.10%). Among adolescents with depression, those who also had suicidal ideation reported an average sleep quality score of 3.62 (score range 1–6, higher scores indicating better sleep quality, SD = 1.060), compared to 4.11 (SD = 0.924) among those with depression but no suicidal ideation.

Socio‐demographic differences in suicidal ideation were found for age and socio‐economic status, but only age (OR = 1.279 [1.075‐1.521]) remained statistically significant in the model adjusted for depression (Table 2, “Model adjusted for depression”).

**Table 2:** Logistic regression: Associations between weekday sleep duration, sleep quality, and suicidal ideation (*n* = 4433).

	Model unadjusted for depression				Model adjusted for depression			
Variable	b	SE	p	OR (95% CI)	b	SE	p	OR (95% CI)
Gender (girls)	0.354	0.251	0.1578	1.425 (0.872– 2.330)	−0.048	0.256	0.8506	0.953 (0.577–1.573)
Age	0.255*	0.085	0.0028	1.290 (1.092–1.525)	0.246*	0.089	0.0055	1.279 (1.075–1.521)
Socio‐economic status	−0.269*	0.076	0.0004	0.764 (0.659–0.886)	−0.136	0.075	0.0697	0.873 (0.754–1.011)
Sleep duration weekdays	−0.276*	0.095	0.0038	0.759 (0.629–0.915)	−0.211*	0.095	0.0267	0.810 (0.672–0.976)
Sleep quality	−0.646*	0.092	< 0.0001	0.524 (0.438–0.628)	−0.358*	0.098	0.0003	0.699 (0.577–0.847)
Depression	—	—	—	—	2.024*	0.236	< 0.0001	7.568 (4.764–12.024)

*Notes:* b = beta coefficient. SE = cluster‐robust standard error. OR = odds ratio. CI = confidence interval. All continuous variables were z‐standardized for the regression model. Therefore, odds ratios indicate the change in the odds of depression that is associated with a one standard deviation increase in the predictor variable.

* Statistically significant at *p* < 0.01

The logistic regression analysis showed that worse sleep quality (OR = 0.524 [0.438–0.628]) and shorter weekday sleep duration (OR = 0.759 [0.629–0.915]) were significantly associated with increased odds of suicidal ideation in the model adjusted for sociodemographic variables, but not depression (Table 2, “Model unadjusted for depression”).

Importantly, the effects of sleep on suicidal ideation remained statistically significant after adjusting for the effect of depression. While depression has by far the largest effect on suicidal ideation (OR = 7.568 [4.764–12.024]), shorter weekday sleep duration (OR = 0.810 [0.672–0.976]) and worse sleep quality (OR = 0.699 [0.577–0.847]) still increase the risk of suicidal ideation. The effect size of sleep duration (OR = 0.810) corresponds to 72 min longer sleep duration and for sleep quality (OR = 0.699) to 0.84 score points (range 1–6).

## 4 Discussion and Conclusion

In this study, suicidal ideation in adolescents was associated with shorter weekday sleep duration, which is in line with previous studies (Sarchiapone et al. 2014; Winsler et al. 2015). Our findings also confirm previous research showing that problems relating to the self‐perceived quality of sleep, such as difficulty initiating sleep, sleep disturbance, and early awakenings, carry an increased risk for suicidal ideation in adolescents (Blank et al. 2015; J.‐W. Liu et al. 2019; Wong, Brower, and Craun 2016).

Given that sleep‐related problems have been linked to psychopathologies in adolescents, such as depression (Lemke et al. 2023), and that depression is linked to suicidal ideation, the primary focus of this study was to investigate whether sleep had any *direct* association with suicidal ideation. The results of this study show indeed that both sleep quality and sleep duration were associated with suicidal ideation even after adjusting for depression. Previous studies reported mixed results regarding the direct effect of sleep on suicidal ideation, with some studies suggesting that the relationship between sleep‐related problems, such as short sleep duration and sleep disturbance, and suicidal ideation disappears after adjusting for depression (X. Liu 2004; Chung et al. 2014). It is possible that these studies were underpowered to identify a direct sleep‐effect as they both had relatively small samples (1400 and 600 respectively). Wong, Brower and Craun (2016) did find similar results to the one reported in our study, however, the sleep‐related variable that was associated to suicidality was “difficulties falling and staying asleep”. This variable is of course one of the components of sleep quality, but not necessarily related to duration.

The model proposed by Goldstein and Franzen (2022) that framed the present study suggests several potential mechanisms underlying the associations between sleep and suicidal outcomes in adolescents. These may involve both proximal risk factors for suicidality, such as affective and behavioral dysregulation, and more distal risk factors such as psychopathologies.

Impaired problem‐solving may be one such proximal factor. At high levels of stress and during critical life events, adolescents with impaired problem‐solving skills appear to be at higher risk for suicidal ideation (Grover et al. 2009); problem‐solving in turn has been shown to be reduced in adolescents who experience problems relating to their sleep (Palmer et al. 2018; Kearns et al. 2020). Another proximal factor that may be involved is emotion dysregulation. i.e., difficulty managing and responding to emotional experiences. For example, sleep disturbance has been shown to increase engagement in impulsive behaviors (Bauducco, Salihovic, and Boersma 2019), maladaptive emotion regulation strategies (Palmer et al. 2018), and altered affective reactivity to interpersonal events (Hamilton et al. 2023). Emotion dysregulation is also a risk factor for suicidal ideation (Brausch, Clapham, and Littlefield 2022). Finally, sleep problems may also influence risk for suicidal ideation through exaggerated stress response and HPA axis activation (Goldstein and Franzen 2022).

Some of the proximal factors potentially linking sleep and suicidal ideation may also be interacting with distal factors involved in the risk for suicidal outcomes, such as psychopathologies (Goldstein and Franzen 2022). For example, sleep‐related problems could be associated with depression through impaired affective and behavioral regulation, such as maladaptive coping strategies (e.g., avoidance, rumination which are commonly associated with lack of sleep, Palmer et al. 2018), and through these distal factors might increase the risk of suicidal ideation. However, our results indicate that the association between sleep and suicidal ideation is likely to involve other pathways than just those related to depression, such as the proximal factors described above.

One limitation of the present study is that the findings are based on cross‐sectional data. As described above, several mechanisms have been suggested to be involved in the associations between suicidal ideation and sleep, often with plausible bi‐directional pathways and interactions of several proximal and distal factors of suicidal outcomes. More longitudinal analyses, especially among adolescent populations, are needed to disentangle the causal mechanisms and temporal patterns of these relationships, for example with regards to the role of proximal factors relating to executive functioning and emotion regulation. Our study suggests that sleep‐related problems are associated with suicidal ideation beyond the effect of depression and that it might be worthwhile to investigate this direct relationship in‐depth using longitudinal models with measures pertaining to putative mechanisms such as emotion dysregulation. In addition, studies should focus on investigating how the sleep‐suicidal ideation associations may differ for short‐term compared to long‐term time predictions as well as for other outcomes of suicidal thoughts and behaviors such as suicide plans and suicide attempts.

Findings from this study highlight the importance of promoting sleep among adolescents, as sleep is a modifiable risk factor for suicidal ideation and provides an entry point for suicide prevention interventions. Interventions should target both duration of sleep on weekends as well as young people's subjective experience of their quality of sleep. There are several effective interventions available that aim at improving adolescents' sleep, including clinical interventions such as Cognitive‐behavioral therapy for insomnia (CBT‐I) (Blake et al. 2017) and universal, structural interventions that aim at creating environments that facilitate healthy sleep habits, such as delaying school start times (Yip et al. 2022). Findings from this study can inform priority setting and planning of public health and suicide‐preventive strategies and policies and can hopefully contribute to an integration of sleep health among young people as one component for comprehensive suicide prevention strategies.

## Ethics Statement

The study was conducted in accordance with the Declaration of Helsinki and was ethically approved by the Regional Ethics Committee in Stockholm (Dnr.: 2016/2175‐31/5). Written consent was obtained from participants, or their parent/guardian if under the age of 15. We have no ethical permission to share original raw data.

## Conflicts of Interest

The authors declare no conflicts of interest.

## Supporting information

Supporting information.

## Data Availability

The data that support the findings of this study are available on request from the corresponding author. The data are not publicly available due to privacy or ethical restrictions. The data that support the findings of this study are available on request from the corresponding author. The data are not publicly available due to ethical restrictions.
