# Supplementary material for: Sleep Duration and Quality in Adolescents: Associations With Suicidal Ideation
Source: J Adolesc. 2025 Jan 25;97(4):1113–20. doi: 10.1002/jad.12473 (PMC12128910; doi:10.1002/jad.12473)
Supplement: Supplementary file 1 — Supporting information. [file JAD-97-1113-s001.docx]

**Appendices**

**Appendix 1**

**Logistic regression model including interaction effects between sleep variables (sleep duration, sleep quality) and depression.**

|  | **Model including interaction effects** | | | |
| --- | --- | --- | --- | --- |
| **Variable** | **b** | **SE** | **p** | **OR (95% CI)** |
| **Gender (girls)** | -0.04928 | 0.233 | 0.8327 | 0.952 (0.607 – 1.519) |
| **Age** | 0.24602 | 0.102 | 0.0155* | 1.279 (1.049 – 1.563) |
| **SES** | -0.13471 | 0.07753 | 0.0823 | 0.874 (0.753 – 1.020) |
| **Sleep duration weekdays** | -0.30458 | 0.21719 | 0.1608 | 0.737 (0.492 – 1.152) |
| **Sleep quality** | -0.41815 | 0.24931 | 0.0935 | 0.658 (0.416 – 1.105) |
| **Depression** | 2.07312 | 0.29195 | <.0001* | 7.950 (4.530 – 14.310) |
| **Sleep duration x depression** | 0.11109 | 0.23709 | 0.6394 | 1.117 (0.691 – 1.749) |
| **Sleep quality x depression** | 0.06942 | 0.26847 | 0.7960 | 1.072 (0.618 – 1.771) |

*Notes:* Associations between weekday sleep duration, sleep quality, and suicidal ideation (n=4433).
b = beta coefficient. SE = standard error. OR = odds ratio. CI = confidence interval.
All continuous variables were z-standardized for the regression model. Therefore, odds ratios indicate the change in the odds of depression that is associated with a one standard deviation increase in the predictor variable.

*Statistically significant at p<.01

**Appendix 2**

**Multilevel logistic regression model (accounting for clustering by schools): Associations between weekday sleep duration, sleep quality, and suicidal ideation (*n*=4433).**

|  | **Model unadjusted for depression** | | | | **Model adjusted for depression** | | | |
| --- | --- | --- | --- | --- | --- | --- | --- | --- |
| **Random effects** | **σ^2^** | **SD** |  |  | **σ^2^** | **SD** |  |  |
| **School factor (intercept)** | 0.3351 | 0.5788 |  |  | 0.4287 | 0.6548 |  |  |
| **Fixed effects** | **b** | **SE** | **p** | **OR (95% CI)** | **b** | **SE** | **p** | **OR (95% CI)** |
| **Gender (girls)** | 0.346 | 0.228 | 0.129 | 1.414 (0.904-2.210) | -0.029 | 0.238 | 0.9033 | 0.971 (0.609-1.550) |
| **Age** | 0.262* | 0.103 | 0.0107 | 1.300 (1.063-1.590) | 0.243* | 0.105 | 0.0204 | 1.276 (1.038-1.567) |
| **SES** | -0.269* | 0.078 | 0.0006 | 0.764 (0.655-0.891) | -0.132 | 0.081 | 0.1019 | 0.876 (0.748-1.026) |
| **Sleep duration weekdays** | -0.288* | 0.091 | 0.0016 | 0.750 (0.627-0.897) | -0.219* | 0.092 | 0.0179 | 0.804 (0.671-0.963) |
| **Sleep quality** | -0.644* | 0.091 | <.0001 | 0.525 (0.440-0.627) | -0.348* | 0.098 | 0.0004 | 0.706 (0.582-0.856) |
| **Depression** | - | - | - | - | 2.077* | 0.285 | <.00010. | 7.982 (4.568-13.948) |

*Notes:* Random-intercept model with a random intercept for schools (n=116).
σ^2^ = variance. SD = standard deviation. b = beta coefficient. SE = standard error. OR = odds ratio. CI = confidence interval.
All continuous variables were z-standardized for the regression model. Therefore, odds ratios indicate the change in the odds of depression that is associated with a one standard deviation increase in the predictor variable.

*Statistically significant at p<.01
